# The Role and Clinical Significance of miR‐484 in the Regulation of SGLT2 in Diabetic Nephropathy

**DOI:** 10.1155/ije/8082721

**Published:** 2026-01-13

**Authors:** Wendi Zhao, Weihua Sun, Qingqing Yang, Li Xue, Chenchen Wu

**Affiliations:** ^1^ Department of Endocrinology, The First Affiliated Hospital of Bengbu Medical University, Bengbu, 233000, China, bbmc.edu.cn

**Keywords:** diabetic nephropathy, hyperglycemia, miR-484, SGLT2

## Abstract

**Background:**

Diabetic nephropathy (DN), a severe complication of Type 2 diabetes mellitus (T2DM), is the primary reason of end‐stage kidney disease (ESKD) and is closely associated with an elevated cardiovascular risk. While miR‐484 has been implicated in diabetes, its specific role in DN remains to be elucidated.

**Objective:**

To analyze miR‐484 expression in DN and its association with clinicopathological parameters, as well as to elucidate its molecular regulation of Sodium–glucose cotransporter protein 2 (SGLT2), providing evidence for the early diagnosis of DN and miR‐484/SGLT2‐targeted therapies.

**Method:**

Clinical data were collected from healthy controls and T2DM patients. Total RNA was extracted for real‐time PCR analysis. ROC curves, Pearson correlation, and logistic regression evaluated their clinical value. High glucose (30 mM)–treated HK‐2 cell models and transfections with miR‐484 mimics/inhibitors assessed cell proliferation, oxidative stress (MDA/SOD), inflammation (TNF‐α/IL‐6/IL‐1β) using cell counting kit‐8 (CCK‐8) and ELISA, and SGLT2 targeting via dual‐luciferase assays.

**Results:**

DN patients exhibited lower serum miR‐484 levels compared to controls and T2DM, negatively correlating with HbA1c and ACR, and positively correlating with eGFR. miR‐484 was an independent risk factor for DN demonstrating high diagnostic sensitivity. High glucose downregulated miR‐484 in HK‐2 cells, inducing proliferation inhibition, oxidative stress, and inflammation; all of which were reversed by miR‐484 mimics. Dual‐luciferase assays confirmed that miR‐484 directly targets the 3′UTR of SGLT2 to suppress its expression.

**Conclusion:**

The miR‐484/SGLT2 axis is key to DN pathogenesis. miR‐484 serum levels reflect DN severity and serve as a potential biomarker. Targeting SGLT2 via miR‐484 offers new therapeutic strategies for DN by mitigating glucose reabsorption, oxidative stress, and inflammation.

## 1. Introduction

Diabetic nephropathy (DN) is a heterogeneous disease characterized by albuminuria and reduced eGFR in diabetic patients [1]. DN plays a critical role in the increased morbidity and mortality associated with Type 2 diabetes mellitus (T2DM) [2]. Globally, DN is now the primary cause of end‐stage kidney disease (ESKD) and is closely linked to an elevated cardiovascular (CV) risk [1]. The pathogenesis of DN is complex, with hyperglycemia serving as a central driver that promotes disease progression through mechanisms such as inflammation, oxidative stress, and apoptosis of renal tubular epithelial cells [3–5]. Currently, clinical practice primarily relies on urine albumin testing and renal function assessment for diagnosis; however, these methods are prone to underdiagnosis [6]. Therefore, the identification of novel biomarkers is essential for early detection, prediction of disease progression, and the implementation of precision medicine.

MicroRNAs (miRNAs), which act as crucial regulators of gene expression, are essential for the development and complications of DN [7, 8]. Previous studies have demonstrated that elevated levels of miR‐21 increase the expression of PI3K and Akt, stimulate the growth of diabetic mouse podocytes, and enhance 24‐h urinary albumin excretion, thereby exacerbating glomerular filtration dysfunction [9]. In addition, the miR‐27a‐3p/TMBIM6 axis influences DN progression by modulating endoplasmic reticulum stress (ER stress) [10]. miR‐484 functions as a key regulatory element in the development of common diseases [11]. Several studies have shown that miR‐484 is significantly downregulated in pancreatic *β*‐cells under high glucose (HG) conditions, and its higher levels exhibit protective effects against diabetes and are associated with insulin resistance–related metabolites, suggesting its close relationship with diabetes [12–14]. However, the expression levels of miR‐484 and the pathophysiological role of miR‐484 in DN patients are still not well understood.

Sodium–glucose cotransporter protein 2 (SGLT2) plays a pivotal role in the pathogenesis of DN by mediating proximal tubular glucose reabsorption [15]. Its overactivation exacerbates glomerular hyperfiltration, tubular epithelial cell injury, and metabolic disorders. SGLT2 inhibitors have been shown to slow the progression of DN through multiple mechanisms and represent a novel class of drugs with established nephroprotective effects following RAAS blockers [16]. Previous studies have demonstrated that SGLT2 inhibitors reduce proximal tubular reabsorption of sodium and glucose, normalize glomerular feedback signaling, and decrease GFR [17]. Clinical studies indicate that SGLT2 inhibitors induce metabolic remodeling by promoting urinary glucose excretion, reducing free fatty acid (FFA) levels, and improving insulin resistance, thereby attenuating systemic and renal local inflammatory responses as well as oxidative stress [18]. Furthermore, SGLT2 inhibitors significantly lower the incidence of ESKD, delay renal function deterioration, and reduce CV mortality risk in patients with DN [19]. Nevertheless, although there is increasing research on SGLT2, the exact molecular mechanism by which miR‐484 regulates SGLT2 is still unclear.

This study systematically investigates the expression profile of miR‐484 in DN and its correlation with clinicopathological parameters. In addition, we aim to elucidate the molecular mechanism by which miR‐484 regulates the downstream gene SGLT2 in the pathogenesis of DN. This study aims to provide experimental evidence and theoretical support for the development of novel intervention strategies based on the miR‐484/SGLT2 axis.

## 2. Materials and Methods

### 2.1. Study Participants and Clinical Data Acquisition

A total of 189 patients with T2DM who were treated at The First Affiliated Hospital of Bengbu Medical University were included in this study. Among them, 103 patients had DN (DN group), while 86 patients did not have DN (T2DM group). In addition, 73 healthy individuals who underwent routine medical check‐ups during the same period were recruited as the control group (HC group). All healthy controls were rigorously screened through detailed medical history interviews to exclude diabetes mellitus and DN. They also had no history of liver disease, autoimmune disease, or infectious disease and had not used antibiotics or corticosteroids within the past month to avoid potential interference with the study results. All participants provided written informed consent after fully understanding the study’s objectives and procedures. The study protocol was reviewed and approved by the Medical Ethics Committee of The First Affiliated Hospital of Bengbu Medical University and strictly followed the ethical guidelines stated in the Declaration of Helsinki.

Clinical data for all study subjects were collected, including age, gender, body mass index (BMI), systolic blood pressure (SBP), and diastolic blood pressure (DBP). After a 10‐h overnight fast, 10 mL of venous blood was drawn from the upper limbs for measurement of glycosylated hemoglobin (HbA1c), fasting blood glucose (FBG), and 2‐h postprandial blood glucose levels during an oral glucose tolerance test (2h‐OGTT). eGFR and urine albumin‐to‐creatinine ratio (ACR) were also assessed concurrently. All laboratory analyses were conducted according to standardized protocols to guarantee the accuracy and reliability of the data.

### 2.2. Extraction of Serum and Total RNA From Cells

We collected 5 mL of venous blood from subjects in a fasting state, allowed it to clot at room temperature for 30 min, and then centrifuged at 3,000 × g for 10 min to separate the serum. 200 μL of the serum was transferred to a new tube and the MolPure Serum/Plasma miRNA Extraction Kit (9332ES50, Yeasen) was used to isolate the total RNA strictly following the manufacturer’s protocol.

For cellular samples, we employed the Cell/Tissue miRNA Extraction Kit (19331ES50, Yeasen) and FastPure Cell/Tissue Total RNA Isolation Kit (RC101‐01, Vazyme), following the lysis, binding, washing, and elution steps as described in the protocol to complete total RNA extraction. After a brief centrifugation, all RNA samples extracted were promptly flash‐frozen in liquid nitrogen and stored at −80°C for further analysis.

### 2.3. Real‐Time Fluorescent Quantitative PCR (RT‐qPCR)

The cDNA was generated through reverse transcription using the miRNA cDNA First Strand Synthesis Kit (with gDNA removal reagent, stem‐and‐loop method) (AG11745, Agbio) and HiScript II Q RT SuperMix for qPCR (+gDNA wiper, R223‐01, Vazyme). qPCR was performed using the miRNA Unimodal SYBR qPCR Master Mix (MQ102, Vazyme) and ChamQ SYBR qPCR Master Mix (Q311‐02, Vazyme).

The levels of miR‐484 expression in serum and cells were measured using U6 as the internal control. The expression levels of SGLT2 in cells were determined with GADPH as the internal reference. Each sample included three technical replicates, and the relative expression levels were computed using the 2^^ΔΔCt^ method.

### 2.4. Cell Culture and HG‐induced Model Construction

Human renal proximal tubular epithelial cells (HK‐2) were obtained from the National Center for the Preservation of Certified Cell Cultures in Shanghai, China. Cells were routinely cultured in low‐glucose DMEM medium (glucose concentration 5.5 mM, 11885084, Thermo Fisher) supplemented with 10% fetal bovine serum (A5256701, Thermo Fisher) and 1% penicillin–streptomycin double antibiotic solution and maintained in a constant‐temperature and humidity incubator at 37°C with 5% CO_2_. When the cells reached 80% confluence, they were passaged using 0.25% trypsin‐EDTA solution, and logarithmic growth phase cells were collected for experiments.

Cells were seeded into 6‐well plates at a density of 1 × 10^5^ cells/well and cultured for 12 h to ensure complete adherence. The original medium was then removed, and the cells were starved in serum‐free DMEM medium for 2 h to eliminate serum interference. Subsequently, the medium was replaced with DMEM containing different glucose concentrations: (1) Normal glucose control group (NG group, 5.5 mM glucose) and (2) HG treatment group (HG group, 30 mM glucose). Three wells were set for each group, and cell samples were gathered following 24 h of culture for subsequent miRNA expression and functional studies.

### 2.5. Cell Transfection

HK‐2 cells were plated in 6‐well plates and maintained overnight at 37°C in a 5% CO_2_ incubator until they reached 70%–80% confluence. Two hours before transfection, the culture medium was exchanged with serum‐free DMEM to synchronize the cell cycle. The miR‐484 mimic or inhibitor, together with their corresponding negative controls, was introduced into the cells using Lipofectamine 3000 (L3000015, Invitrogen) following the manufacturer’s protocol. Following 24 h of transfection, the medium was discarded and replaced with either HG medium (30 mM glucose) or NG medium (5.5 mM glucose), and the cells were cultured for an additional 24 h. Thereafter, the cells were collected for subsequent analysis.

### 2.6. Cell Proliferation Ability Detection

HK‐2 cells were transfected with either miR‐484 mimic or inhibitor and subsequently exposed to HG conditions. These cells were then plated into 96‐well plates, with five replicates for each group. Once the cells had adhered to the plate surface, their viability was evaluated at multiple time points: 0 h, 24 h, 48 h, and 72 h post‐culture. To perform the assessment, 10 μL of cell counting kit‐8 (CCK‐8) (C0038, Beyotime) solution was added to each well, followed by gentle mixing to ensure homogeneity. The plates were incubated at 37°C under 5% CO_2_ for 1 h. The proliferative capacity of the cells was determined by measuring the optical density (OD) of each well at 450 nm using a microplate reader. This procedure was conducted in three independent experiments to ensure reliability.

### 2.7. Flow Cytometry Analysis

Following centrifugation for cell collection, cells were stained using the Annexin V‐FITC/PI Apoptosis Detection Kit (40302ES50, Yeasen), and apoptosis rates in each group were quantitatively analyzed by flow cytometry.

### 2.8. Enzyme‐Linked Immunosorbent Assay (ELISA)

Intracellular oxidative stress markers, including malondialdehyde (MDA) and superoxide dismutase (SOD), as well as inflammatory cytokines such as IL‐1β, IL‐6, and TNF‐α, were quantified using ELISA. Specific detection kits were utilized for these measurements: MDA assay kit, SOD assay kit, human IL‐1β ELISA kit, human IL‐6 ELISA kit, and human TNF‐α ELISA kit (all provided by Thermo Fisher). For each analyte, three technical replicates were established to enhance precision. To ensure the reliability of the results, all experiments were conducted independently on three independent occasions.

### 2.9. Dual‐Luciferase Reporter Gene Assay

Using the bioinformatics database TargetScan Human, SGLT2 was identified as a potential target gene of miR‐484. Luciferase reporter plasmids containing the wild‐type (SGLT2‐WT) and mutant (SGLT2‐Mut) sequences of its 3′UTR were constructed. The plasmids were co‐introduced along with miR‐484 mimics, inhibitors, or their respective negative controls into HK‐2 cells. 48 h later, the luciferase activity was detected using the dual‐luciferase reporter assay system (Promega) and standardized to the Renilla luciferase activity as an internal control. This experiment was performed independently three times, with six technical replicates for each group.

### 2.10. Immunoblotting Assay

Total protein from the collected cells was extracted using radioimmunoprecipitation assay (RIPA) lysis buffer (Solarbio, Beijing, China). Subsequently, an immunoblot assay was performed as previously described [20].

### 2.11. Statistical Analysis

Statistical analyses and graphical plotting were performed using SPSS and GraphPad Prism 7.0. The data are presented as mean ± standard deviation (SD). Statistical analyses were conducted using a two‐tailed *t*‐test for pairwise comparisons between the two groups and one‐way ANOVA followed by Tukey’s post hoc test for comparisons among multiple groups, and repeated‐measures ANOVA for evaluating data collected at various time points between the two groups. All experiments were independently repeated at least three times. Receiver operating characteristic (ROC) curves were generated to evaluate the diagnostic capability of miR‐484 for DN. The Pearson correlation coefficient was used to analyze the relationship between miR‐484 expression levels and DN‐related clinicopathological parameters. Logistic regression analysis was conducted to identify risk factors for DN in T2DM patients. Statistical significance was defined as a *p* value < 0.05.

## 3. Results

### 3.1. The Expression and Clinical Significance of miR‐484 in T2DM

In this study, 103 patients with DN, 86 patients with T2DM, and 73 healthy subjects were enrolled. Table 1 summarizes the baseline characteristics and biochemical parameters of each group. The findings indicated that no significant differences were observed in age, sex, prevalence of hypertension (HTN), or BMI among the three groups (*p* > 0.05). However, notable differences were detected in HbA1c, FBG, 2h‐OGTT, eGFR, and urine ACR (*p* < 0.05).

**Table 1:** Comparison of clinical data between the HC group, T2DM group, and DN group.

Statistical indicators	HC	T2DM	DN	*p* value
Age	52.95 ± 8.048	53.77 ± 6.232	52.86 ± 6.942	0.643
Gender (male/female)	44/29	49/37	64/39	0.770
HTN (yes/no)	7/66	13/73	19/84	0.270
BMI (kg/m^2^)	22.68 ± 1.114	22.80 ± 1.118	22.75 ± 1.659	0.872
HbA1c (%)	4.861 ± 0.568	6.768 ± 0.821	7.320 ± 0.826	< 0.001
FBG (mmol/mL)	6.182 ± 0.735	7.056 ± 0.893	7.462 ± 0.927	< 0.001
2h‐OGTT (mmol/mL)	9.421 ± 1.059	11.16 ± 1.313	11.35 ± 1.653	< 0.001
eGFR (mL/min/1.73 m2)	99.40 ± 3.637	92.18 ± 9.129	66.11 ± 9.356	< 0.001
Urinary ACR (mg/g)	/	22.69 ± 3.106	172.2 ± 36.990	< 0.001

Serum miR‐484 levels were significantly lower in both T2DM patients and DN patients compared to healthy controls (*p* < 0.05, Figure 1(a)). Further analysis using ROC curves revealed that miR‐484 effectively distinguished between healthy individuals and T2DM patients, with an area under the curve (AUC) of 0.853, a sensitivity of 79.07%, and a specificity of 78.08% (Figure 1(b)). For differentiating between T2DM patients and DN patients, miR‐484 exhibited an AUC of 0.829, a sensitivity of 80.58%, and a specificity of 74.42% (Figure 1(c)).

Figure 1The differential expression and diagnostic significance of serum miR‐484 in healthy subjects, individuals with T2DM, and those with DN. (a) Comparison of serum miR‐484 expression levels among the healthy group, T2DM group and DN group, HC versus T2DM ^∗∗∗^ *p* < 0.001, and HC versus DN ###*p* < 0.001. (b) ROC curve analysis of the diagnostic value of miR‐484 for the healthy group and the T2DM group, *p* < 0.001. (c) ROC curve analysis of the diagnostic value of miR‐484 for the T2DM group and the DN group, *p* < 0.001.

**Figure:**
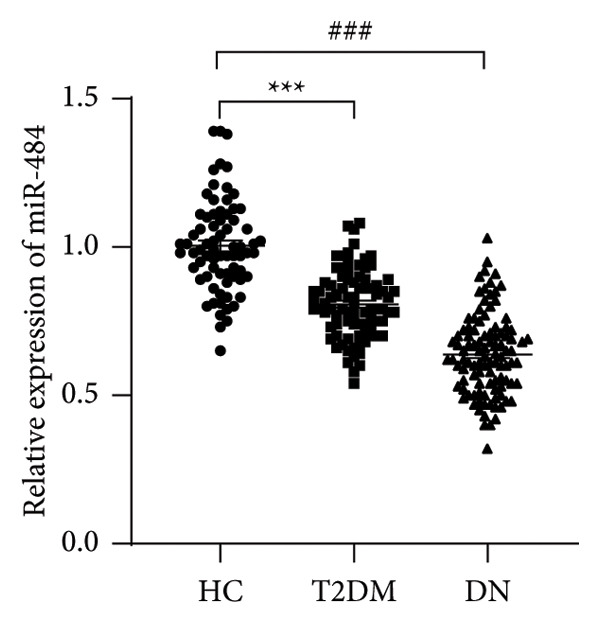
(a)

**Figure:**
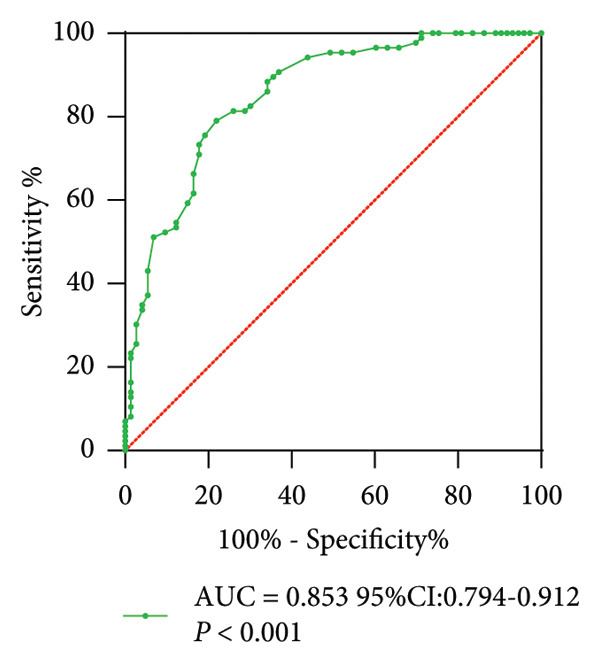
(b)

**Figure:**
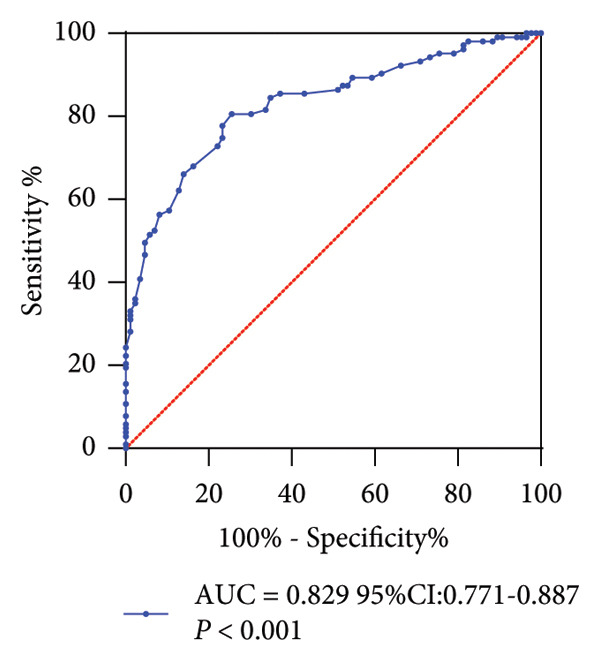
(c)

### 3.2. Serum miR‐484 Expression is Significantly Lower in T2DM Patients With Abnormal Glucose Metabolism and Renal Dysfunction and Serves as an Independent Risk Factor for DN

Pearson correlation analysis revealed that the serum miR‐484 expression level was significantly associated with multiple clinical biochemical indicators (*p* < 0.001, Table 2). Specifically, miR‐484 exhibited a significant inverse association with HbA1c (*r* = −0.669), FBG (*r* = −0.666), 2h‐OGTT (*r* = −0.630), and ACR (*r* = −0.599). These results suggest that reduced miR‐484 expression is closely linked to more severe glucose metabolism disorders and proteinuria. Notably, miR‐484 demonstrated a significant positive association with eGFR (*r* = 0.630), suggesting that its expression level may serve as an indicator of renal function.

**Table 2:** Pearson correlation analysis was performed to investigate the correlations between miR‐484 and various clinical indicators in patients.

Indicators	Pearson correlation coefficient	*p* value
HbA1c	−0.669	< 0.001^∗∗∗^
FBG	−0.666	< 0.001^∗∗∗^
2h‐OGTT	−0.630	< 0.001^∗∗∗^
eGFR	0.6301	< 0.001^∗∗∗^
Urinary ACR	−0.599	< 0.001^∗∗∗^

^∗∗∗^indicates *P* < 0.001.

**Figure 2:**
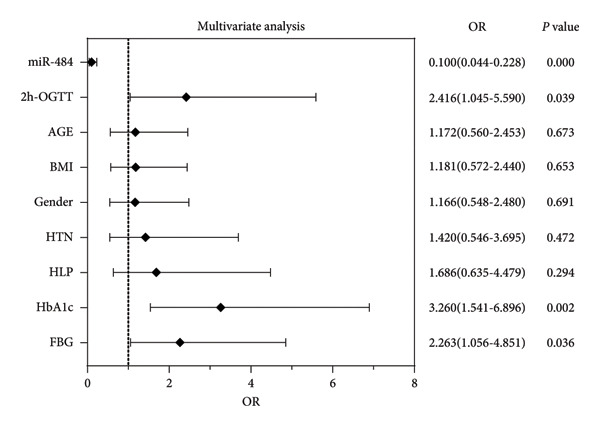
Logistic regression analysis of risk factors associated with DN in T2DM patients.

Furthermore, multifactorial logistic regression analysis revealed that miR‐484 (OR = 0.100, 95% CI: 0.044–0.228, *p* < 0.001), HbA1c (OR = 3.260, 95% CI: 1.541–6.896, *p* = 0.002), FBG (OR = 2.263, 95% CI: 1.056–4.851, *p* = 0.036), and 2h‐OGTT (OR = 2.416, 95% CI: 1.045–5.590, *p* = 0.039) are independent risk factors for the progression of DN in T2DM patients (Figure 2).

### 3.3. miR‐484 Mimics Alleviate the HG‐Induced Suppression of HK‐2 Cell Proliferation, Oxidative Stress, and Inflammatory Reactions

The findings indicated that miR‐484 expression in HK‐2 cells was significantly reduced following HG stimulation. Subsequently, the introduction of miR‐484 mimics into HG‐treated HK‐2 cells restored miR‐484 expression (*p* < 0.01, Figure 3(a)).

Figure 3The impact of HG treatment on miR‐484 expression and functional activity in HK‐2 cells. (a) The expression level of miR‐484 in HK‐2 cells before and after HG treatment was evaluated by PCR, control versus HG ^∗∗∗^ *p* < 0.001, and HG + mimic NC versus HG + miR‐484 mimic ##*p* < 0.01. (b) The influence of different treatments on cell proliferation activity, control versus HG ^∗∗∗^ *p* < 0.001, and HG + mimic NC versus HG + miR‐484 mimic ###*p* < 0.001. (c) Flow cytometry was employed to assess apoptosis in HK‐2 cells across experimental groups, control versus HG ^∗∗∗^ *p* < 0.001, and HG + mimic NC versus HG + miR‐484 mimic ###*p* < 0.001. *n* = 3 independent experiments. All data are presented as mean ± standard deviation.

**Figure:**
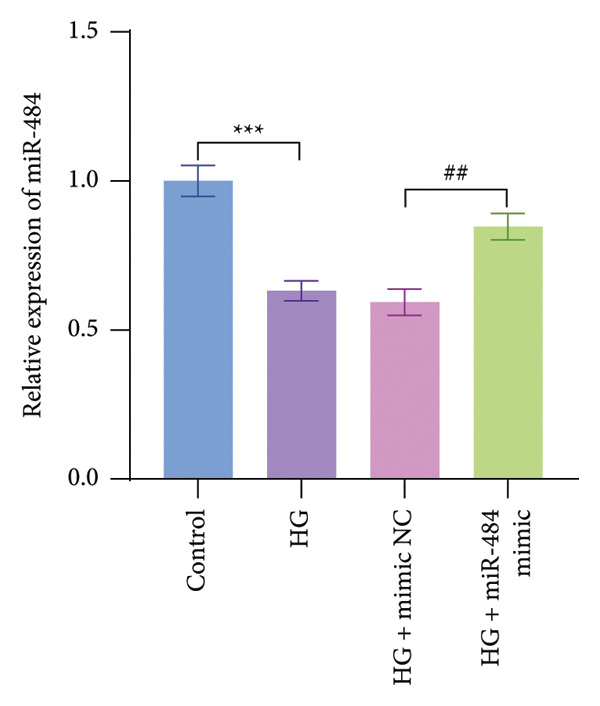
(a)

**Figure:**
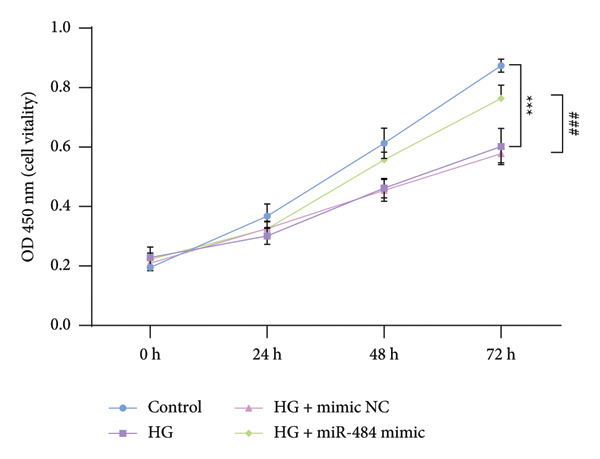
(b)

**Figure:**
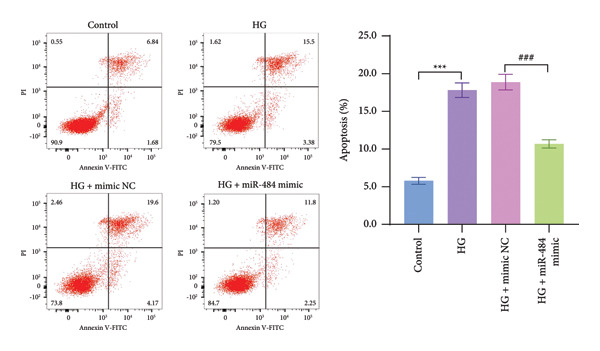
(c)

Further analysis via a cell proliferation assay demonstrated that HG exposure significantly diminished the proliferative capacity of HK‐2 cells. In contrast, miR‐484 mimics were able to safeguard and promote cell proliferation under HG conditions, thereby mitigating the suppressive impact of HG on cell growth (*p* < 0.001, Figure 3(b)).

Further analysis of cell apoptosis demonstrated that HG significantly induces apoptosis in HK‐2 cells, whereas transfection with the miR‐484 mimic effectively suppresses HG‐induced apoptosis in these cells (*p* < 0.001, Figure 3(c)).

### 3.4. Overexpression of miR‐484 Effectively Suppresses HG‐Induced Oxidative Stress and Inflammatory Responses

Regarding oxidative stress markers, HG treatment led to elevated MDA levels and reduced SOD activity within the cells. Upon transfection with miR‐484 mimics, MDA levels declined while SOD levels recovered, indicating that miR‐484 mimics are able to alleviate the oxidative stress caused by HG (*p* < 0.05, Figures 4(a) and 4(b)).

Figure 4The effect of miR‐484 on oxidative stress and inflammatory responses in HK‐2 cells under HG conditions. (a) Changes in the levels of oxidative stress markers MDA, control versus HG ^∗∗∗^ *p* < 0.001, and HG + mimic NC versus HG + miR‐484 mimic ###*p* < 0.001. (b) Changes in the levels of oxidative stress markers SOD, control versus HG ^∗∗∗^ *p* < 0.001, and HG + mimic NC versus HG + miR‐484 mimic ##*p* < 0.01. (c) Expression levels of inflammatory factors IL‐1β, control versus HG ^∗∗∗^ *p* < 0.001, and HG + mimic NC versus HG + miR‐484 mimic ###*p* < 0.001. (d) Expression levels of inflammatory factors IL‐6, control versus HG ^∗∗∗^ *p* < 0.001, and HG + mimic NC versus HG + miR‐484 mimic #*p* < 0.05. (e) Expression levels of inflammatory factors TNF‐α, control versus HG ^∗∗∗^ *p* < 0.001, and HG + mimic NC versus HG + miR‐484 mimic ###*p* < 0.001. *n* = 3 independent experiments. All data are presented as mean ± standard deviation.

**Figure:**
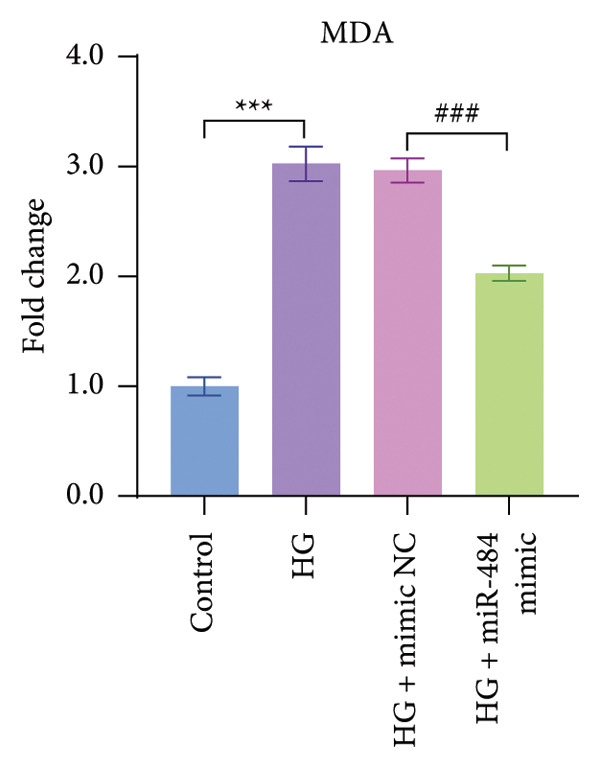
(a)

**Figure:**
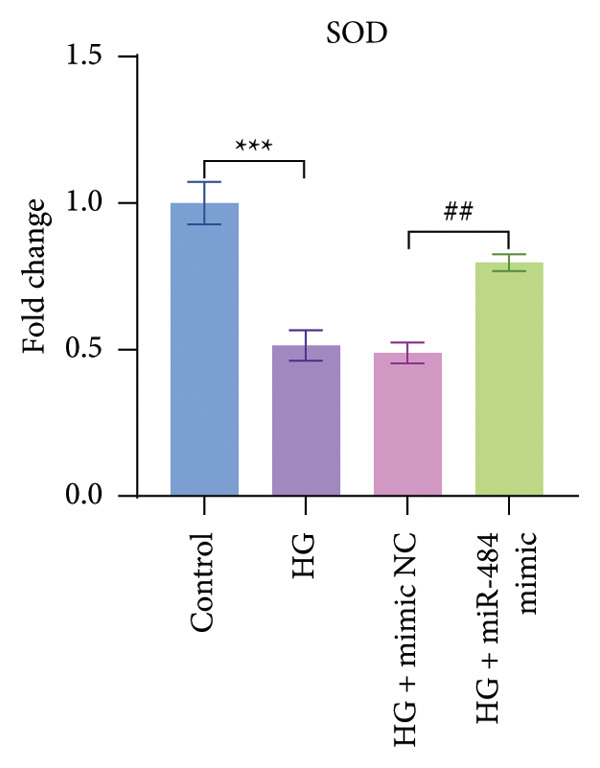
(b)

**Figure:**
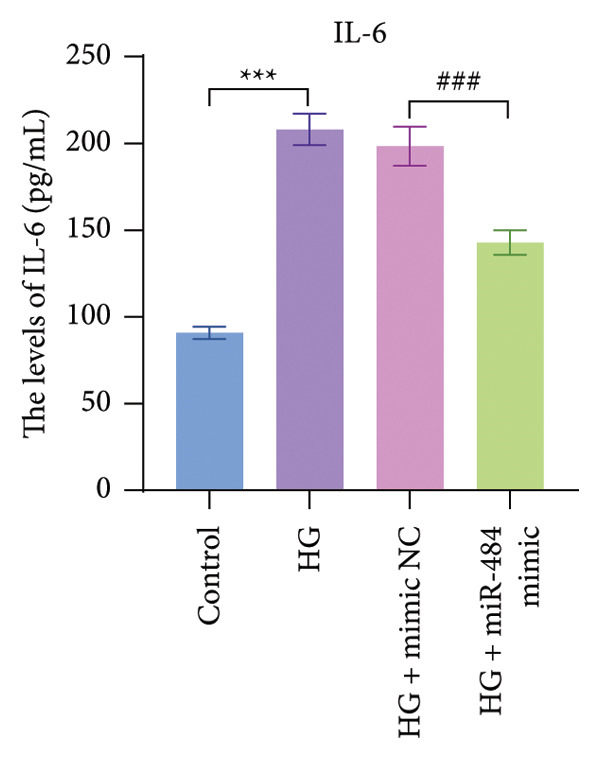
(c)

**Figure:**
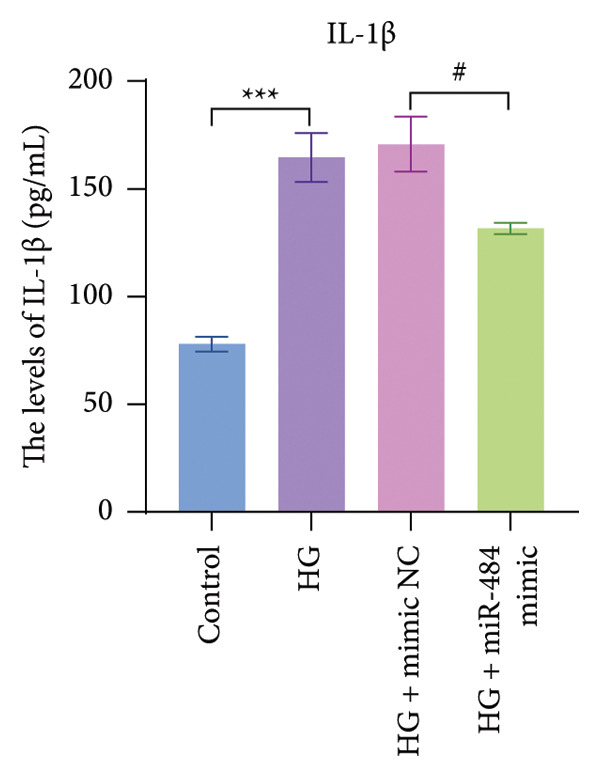
(d)

**Figure:**
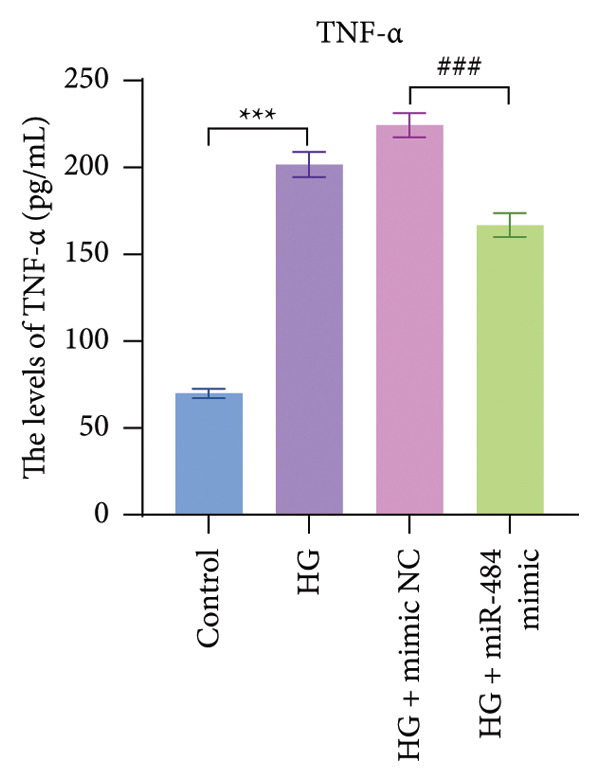
(e)

With respect to inflammation‐related indicators, HG exposure increased the levels of TNF‐α, IL‐6, and IL‐1β, suggesting that HG can elicit inflammatory responses in cells. After transfection with miR‐484 mimics, the levels of these inflammatory cytokines decreased, implying that miR‐484 mimics possess the ability to dampen HG‐induced cellular inflammation (*p* < 0.05, Figures 4(c), 4(d), and 4(e)).

### 3.5. miR‐484 Interacts With SGLT2 Targets and HG Enhances SGLT2 Expression While Being Regulated by miR‐484

By utilizing the online tool TargetScan, SGLT2 was predicted as a possible downstream target of miR‐484 (Figure 5(a)). This prediction was subsequently confirmed through a dual‐luciferase reporter assay. The assay showed that transfecting with miR‐484 mimics markedly reduced the activity of the SGLT2‐WT (*p* < 0.05, Figure 5(b)). In contrast, the SGLT2‐MUT exhibited no significant change in activity upon transfection with miR‐484 mimics (*p* < 0.05, Figure 5(b)). Furthermore, transfection with an miR‐484 inhibitor led to a notable increase in the activity of the SGLT2‐WT vector. These findings provide robust evidence for the direct and specific binding of miR‐484 to the SGLT2 target site.

Figure 5Verification of miR‐484–targeted regulation on SGLT2 and its effects on expression. (a) The TargetScan Human database predicted the potential binding sites of SGLT2 for miR‐484. (b) The effects of miR‐484 mimics and inhibitors on luciferase activity in SGLT2‐WT were assessed using a luciferase assay, mimic NC versus miR‐484 mimic ^∗∗^ *p* < 0.01, and inhibitor NC versus miR‐484 inhibitor ###*p* < 0.001. (c) The relative expression level of SGLT2 mRNA was detected by qPCR, control versus HG ^∗∗^ *p* < 0.01, HG + mimic NC versus HG + miR‐484 mimic #*p* < 0.05, and HG + inhibitor NC versus HG + miR‐484 inhibitor + *p* < 0.05. (d) The expression of SGLT2 protein was analyzed by western blotting with GAPDH as the loading control. Band intensities were quantified using ImageJ software and normalized to GAPDH. (e) The bar chart depicts the relative expression level of SGLT2 protein. Data are presented as mean ± standard deviation. Compared with the control group, the high‐glucose group showed ^∗∗∗^ *p* < 0.001. Compared with the high‐glucose group transfected with mimic negative control, the miR‐484 mimic group exhibited #*p* < 0.05. Similarly, compared with the high‐glucose group transfected with inhibitor negative control, the miR‐484 inhibitor group showed ++*p* < 0.01. *n* = 3 independent experiments.

**Figure:**
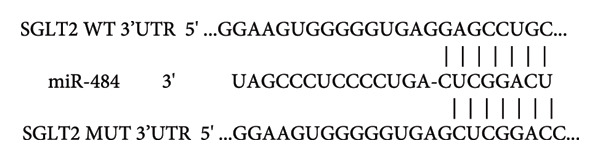
(a)

**Figure:**
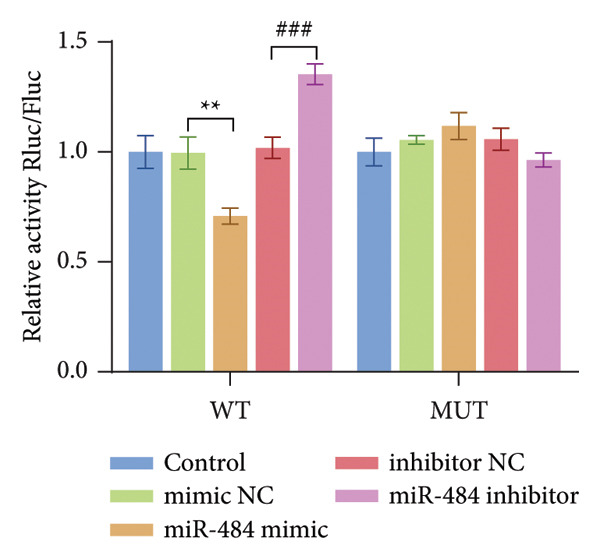
(b)

**Figure:**
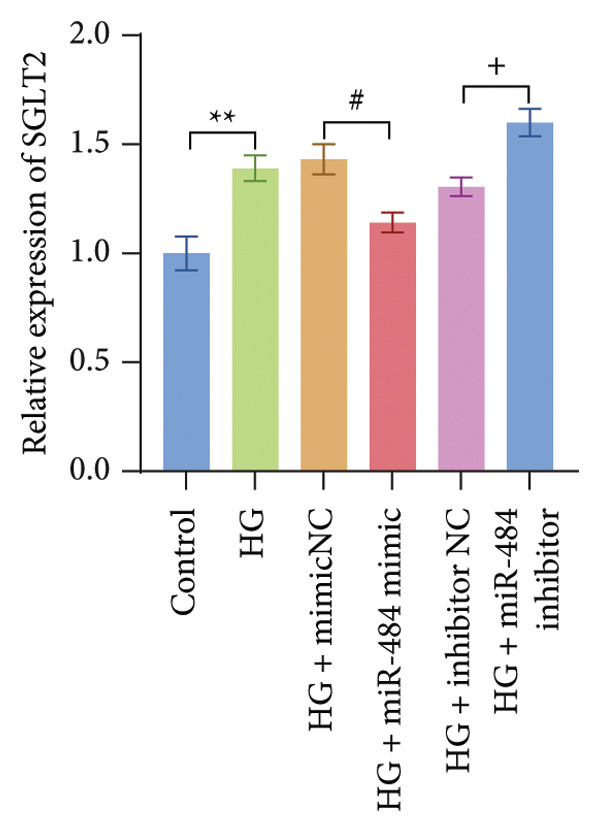
(c)

**Figure:**
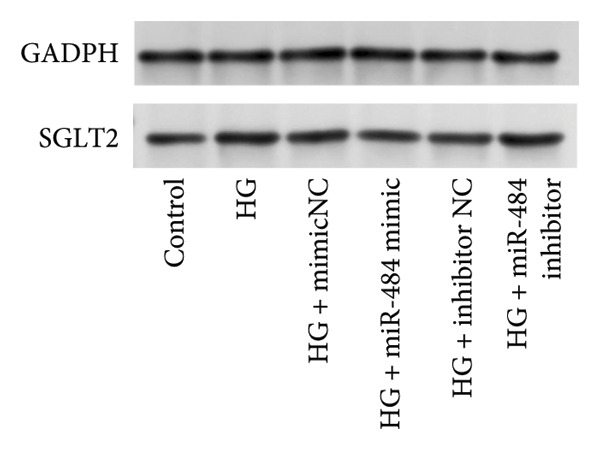
(d)

**Figure:**
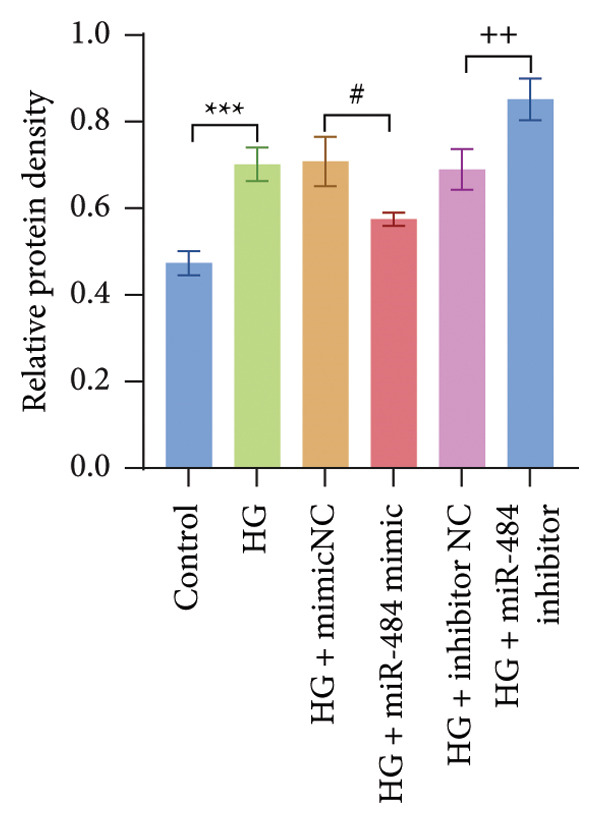
(e)

In addition, qPCR and Western blot experiments were conducted to evaluate the relative expression levels of SGLT2. The qPCR results indicated that treatment with HG resulted in elevated SGLT2 expression compared to the control group, suggesting that a HG environment may stimulate SGLT2 expression. Notably, transfection with miR‐484 mimics suppressed SGLT2 expression under HG conditions, whereas transfection with an miR‐484 inhibitor enhanced SGLT2 expression (*p* < 0.05, Figure 5(c)). Western blot analysis demonstrated that HG treatment significantly increased SGLT2 protein expression; in contrast, transfection with miR‐484 mimics markedly reduced SGLT2 protein levels, whereas transfection with miR‐484 inhibitors led to a significant upregulation of SGLT2 protein expression (*p* < 0.05, Figure 5(d)). These findings are consistent with the mRNA expression data.

## 4. Discussion

DN is among the most serious microvascular complications of T2DM and remains the primary cause of end‐stage renal disease worldwide [21]. Currently, the primary clinical diagnostic methods include urine albumin testing, eGFR, renal ultrasound, CT, MRI, and renal biopsy [22]. However, these approaches are often associated with issues such as underdiagnosis, high cost, and significant risk [23]. Thus, there is an urgent need for the development of novel biomarkers. This study systematically elucidated the expression profile of miR‐484 in DN and its regulatory mechanism on SGLT2 in HG‐induced renal injury through clinical sample analysis, cellular functional experiments, and molecular mechanism validation, offering a new avenue for early warning and intervention in DN.

Several recent studies have demonstrated that miRNAs hold significant potential as biomarkers for the clinical diagnosis of DN [24, 25]. For instance, miR‐223 exhibited reduced expression levels in DN patients, suggesting its potential utility in diagnosing DN [26]. In addition, serum levels of miR‐23a‐3p were found to be decreased in patients with T2DM, with more pronounced reductions as the disease progressed. These findings indicate that miR‐23a‐3p may serve as a marker for the early diagnosis and monitoring of disease progression in T2DN [27]. Meanwhile, low serum expression of miR‐192 was associated with early‐stage DN and facilitated its early prediction. In contrast, elevated levels of miR‐192 in renal tissues and urine might indicate disease progression in advanced DN [28]. The clinical baseline analysis of this study revealed significant differences in glucose metabolism and renal function indices among the three groups (*p* < 0.05), confirming the strong association between DN progression, glucose metabolism disorders, and renal injury. The negative correlation between serum miR‐484 levels and DN severity suggests that miR‐484 can serve as a dual indicator reflecting both glucose metabolism disorders and renal damage. ROC curve analysis further demonstrated that miR‐484 effectively distinguishes healthy individuals from T2DM patients, as well as T2DM patients from those with DN, highlighting its potential as an early diagnostic biomarker for DN. Multivariate logistic regression analysis confirmed that miR‐484 is an independent risk factor for DN development, with predictive efficacy comparable to traditional indicators such as HbA1c and FBG. These findings underscore the clinical significance of miR‐484 in the early identification and diagnosis of DN and provide a critical foundation for the development of noninvasive detection methods and targeted intervention strategies targeting the miR‐484 axis.

Hyperglycemia‐induced apoptosis and oxidative stress damage are acknowledged as key contributors to the development of DN [29]. HG concentrations suppress cell viability and induce mitochondrial fission in HK2 cells [30]. Hyperglycemia can induce mitochondrial damage, activate the collateral glucose metabolism pathway, and enhance the spontaneous glucose response, promoting excessive ROS generation and thereby leading to oxidative stress [31]. Earlier research has demonstrated that miR‐484 is strongly linked to diabetes [12–14]. Therefore, in this study, an HG‐induced HK‐2 cell injury model was established. The findings indicated that miR‐484 expression was substantially reduced in HK‐2 cells under HG conditions. HG markedly suppressed the proliferative activity of HK‐2 cells, induced cell apoptosis, increased the level of MDA, a marker of intracellular oxidative stress, reduced the activity of SOD, and elevated the concentrations of inflammatory cytokines, including TNF‐α, IL‐6, and IL‐1β. After transfection with miR‐484 mimics, these abnormal changes were effectively reversed; cell proliferation was partially restored, and oxidative stress and inflammatory responses were alleviated. These results suggest that miR‐484 serves a crucial protective function in HG‐induced kidney damage in DN, exerting its effects via various mechanisms such as modulating oxidative stress, inflammation, and cell proliferation. This offers direct experimental support for the potential of miR‐484 as a therapeutic target in DN and provides an essential starting point for further exploration of the molecular mechanisms behind miRNA deregulation in DN.

Enhanced sodium–glucose cotransport due to the hyperactivation of SGLT2, a key transporter for glucose reabsorption in the proximal tubule, exacerbates glomerular hyperfiltration via a tubuloglomerular feedback mechanism and induces tubular epithelial cell steatosis with mitochondrial dysfunction [32, 33]. Previous studies have demonstrated that SGLT2 inhibitors modulate metabolism and mitigate the progression of chronic kidney disease by reducing CV risk [34, 35]. In this study, we confirmed that SGLT2 is a target gene of miR‐484 using TargetScan Human prediction and dual‐luciferase reporter assays. The results revealed that miR‐484 specifically binds to the 3′UTR of SGLT2, thereby negatively regulating SGLT2 expression at the posttranscriptional level. HG promotes SGLT2 expression by downregulating miR‐484, which exacerbates the pathogenesis of DN. While previous studies have primarily focused on the clinical application of SGLT2 inhibitors, the present study elucidates the precise regulation of SGLT2 by miR‐484 at the molecular level, providing a theoretical foundation for the design of novel miRNA‐based therapeutic strategies. It is anticipated that regulating endogenous miR‐484 levels could enable dynamic control of SGLT2 expression and improve therapeutic precision.

However, despite the promising results of this study, certain limitations remain. First, the relatively limited sample size might restrict the generalizability of the results to broader populations, and multicenter studies with larger cohorts are required to further validate the diagnostic efficacy of miR‐484. Second, future studies will compare miR‐484 levels in serum and urine to investigate their combined diagnostic value and further clarify the role of miR‐484 in DN diagnosis. Third, the therapeutic efficacy and safety of miR‐484 need to be rigorously evaluated in animal models and clinical trials. Finally, miR‐484 may regulate DN through other uncharacterized targets, which warrant further investigation to comprehensively elucidate its mechanism of action.

In summary, the present study clarified the regulatory function of the miR‐484/SGLT2 axis in DN. We confirmed that miR‐484 serves not only as a potential indicator for the early screening and assessment of DN but also as a therapeutic candidate that improves glucose metabolism and protects renal function by inhibiting SGLT2 expression. These findings provide new insights and are promising candidates for the early detection, risk stratification, and management of DN, thereby advancing precision medicine approaches for DN and potentially improving patient outcomes.

## Ethics Statement

The study was performed in line with the principles of the Declaration of Helsinki. Approval was granted by the Ethics Committee of The First Affiliated Hospital of Bengbu Medical University before the study began. Written informed consent has been obtained from the participants involved.

## Consent

Please see the Ethics Statement.

## Conflicts of Interest

The authors declare no conflicts of interest.

## Funding

The authors did not receive support from any organization for the submitted work.

## Data Availability

Corresponding authors may provide data and materials.
